# Improving US maternal mortality reporting by analyzing literal text on death certificates, United States, 2016–2017

**DOI:** 10.1371/journal.pone.0240701

**Published:** 2020-10-28

**Authors:** Marian F. MacDorman, Marie Thoma, Eugene Declercq

**Affiliations:** 1 Maryland Population Research Center, University of Maryland, College Park, Maryland, United States of America; 2 Department of Family Science, University of Maryland School of Public Health, College Park, Maryland, United States of America; 3 Department of Community Health Sciences, Boston University School of Public Health, Boston, Massachusetts, United States of America; University of Mississippi Medical Center, UNITED STATES

## Abstract

Changes in data collection and processing of US maternal mortality data across states over time have led to inconsistencies in maternal death reporting. Our purpose was to identify possible misclassification of maternal deaths and to apply alternative coding methods to improve specificity of maternal causes. We analyzed 2016–2017 US vital statistics mortality data with cause-of-death literals (actual words written on the death certificate) added. We developed an alternative coding strategy to code the “primary cause of death” defined as the most likely cause that led to death. We recoded deaths with or without literal pregnancy mentions to maternal and non-maternal causes, respectively. Originally coded and recoded data were compared for overall maternal deaths and for a subset of deaths originally coded to ill-defined causes. Among 1691 originally coded maternal deaths, 597 (35.3%) remained a maternal death upon recoding and 1094 (64.7%) were recoded to non-maternal causes. The most common maternal causes were eclampsia and preeclampsia, obstetric embolism, postpartum cardiomyopathy, and obstetric hemorrhage. The most common non-maternal causes were diseases of the circulatory system and cancer, similar to the leading causes of death among all reproductive-age women (excluding injuries). Among 735 records originally coded to ill-defined causes, 94% were recoded to more specific, informative causes from literal text. Eighteen deaths originally coded as non-maternal mentioned pregnancy in the literals and were recoded as maternal deaths. Literal text provides more detailed information on cause of death which is often lost during coding. We found evidence of both underreporting and overreporting of maternal deaths, with possible overreporting predominant. Accurate data is essential for measuring the effectiveness of maternal mortality reduction programs.

## Introduction

Vital statistics data is the foundation of the public health system and provide essential data to monitor public health programs. Maternal mortality is a sentinel indicator of health care quality, however concerns about the quality of US maternal mortality reporting have lingered for decades [1–3]. Vital statistics maternal mortality data is also the primary data source for more sophisticated maternal mortality review systems, such as the Pregnancy Mortality Surveillance System and maternal mortality review committees, and inaccuracies in vital statistics data create problems for these other data systems [4, 5].

A pregnancy checkbox was added to the U.S. standard death certificate in 2003 to improve reporting of maternal deaths [6, 7]. The checkbox asks certifiers to document whether female decedents were: not pregnant within past year; pregnant at time of death; not pregnant, but pregnant within 42 days of death; not pregnant, but pregnant 43 days to 1 year before death; or unknown if pregnant within the past year [1]. The addition of the pregnancy checkbox increased identification of maternal deaths, with states that had adopted it reporting almost twice as many deaths as they had before adoption [2, 3]. Recent validity studies in states that have adopted the checkbox found that it led to over-reporting of maternal deaths, ranging from 21% in a 4 state study [8], to 50% in a Texas study [4]. Studies also found a large and increasing number of maternal deaths coded to ill-defined causes [9].

The ability to conduct large-scale validation studies at the national level are limited, and valuable information from death certificates can be lost during the coding process. Our purpose was to analyze the cause-of-death literals (actual words written on the death certificate) to identify possible under or over-reporting of maternal deaths, and to apply alternative coding methodologies to promote greater specificity in the causes of maternal and late maternal death. Literal text provides detailed information on the diseases and medical circumstances which led to death, including underlying and multiple causes of death, and has been used to examine mortality in studies of sudden infant death syndrome [10], infectious diseases (e.g., influenza) [11], diabetes [11], cancer [12], and drug-related deaths [13–15]. This is the first study to apply analysis of cause-of-death literals to examine US maternal mortality.

## Materials and methods

United States maternal mortality data used for national and international comparisons are based on information reported on death certificates filed in state vital statistics offices, and compiled into national data through the National Vital Statistics System [6]. Physicians, medical examiners or coroners are responsible for completing the medical portion of the death certificate, including the cause of death. Since 1999, cause-of-death data in the United States have been coded according to the International Statistical Classification of Diseases and Related Health Problems, 10^th^ Revision (ICD-10). Maternal deaths are denoted by codes A34, O00-O95, O98-O99, while late maternal deaths are denoted by codes O96-O97 [16]. Unlike maternal deaths, ICD-10 codes for late maternal deaths lack additional specificity in causes of death other than deaths from any obstetric cause occurring 43 days-1 year after delivery (O96) or deaths from sequelae of an obstetric cause (O97).

We used the 2016 and 2017 U.S. multiple cause-of-death data files from the National Center for Health Statistics (NCHS), with cause-of-death literals added. The cause-of-death literals are the actual words written in the cause-of-death section of the death certificate which serve as the basis for assignment of ICD-10 codes and provide much richer detail as to the actual circumstances of death. We created a subset of all possible maternal or late maternal death records to analyze in more detail. We selected all records of females aged 10–54 that contained a maternal condition in the multiple cause-of-death data OR with pregnancy checkbox values of 2 (pregnant), 3 (< = 42 days postpartum), or 4 (43 days-1 year postpartum). This yielded 3,968 records for more detailed analysis.

Although states were slow to adopt the U.S. standard pregnancy question, all but two states (West Virginia and California) were using the standard question by January 2016. West Virginia (with <0.5% of US births) adopted the standard question in mid-2017 and California has a non-standard question which ascertains whether the woman was pregnant or postpartum within 1 year at the time of death. Thus, the California data includes all maternal deaths up to 1 year postpartum, but does not clearly delineate the postpartum time periods (<6 weeks; 6 weeks-1 year). For this analysis, we include information from California and West Virginia to enable us to provide US estimates, and as their exclusion did not appreciably affect the findings.

The United States uses the World Health Organization definition of maternal death: “The death of a woman while pregnant or within 42 days of termination of pregnancy, irrespective of the duration and the site of the pregnancy, from any cause related to or aggravated by the pregnancy or its management, but not from accidental or incidental causes” [17]. Late maternal deaths are those that occur from 43 days-1 year after pregnancy [17]. Direct obstetric deaths are those resulting from obstetric complications of pregnancy, labor and the puerperium, or from interventions, omissions, or incorrect treatment. Indirect obstetric deaths are those resulting from previous existing disease or disease that developed during pregnancy, which was aggravated by the physiologic effects of pregnancy [17].

### Recoding records to the primary cause of death

**Fig 1:**
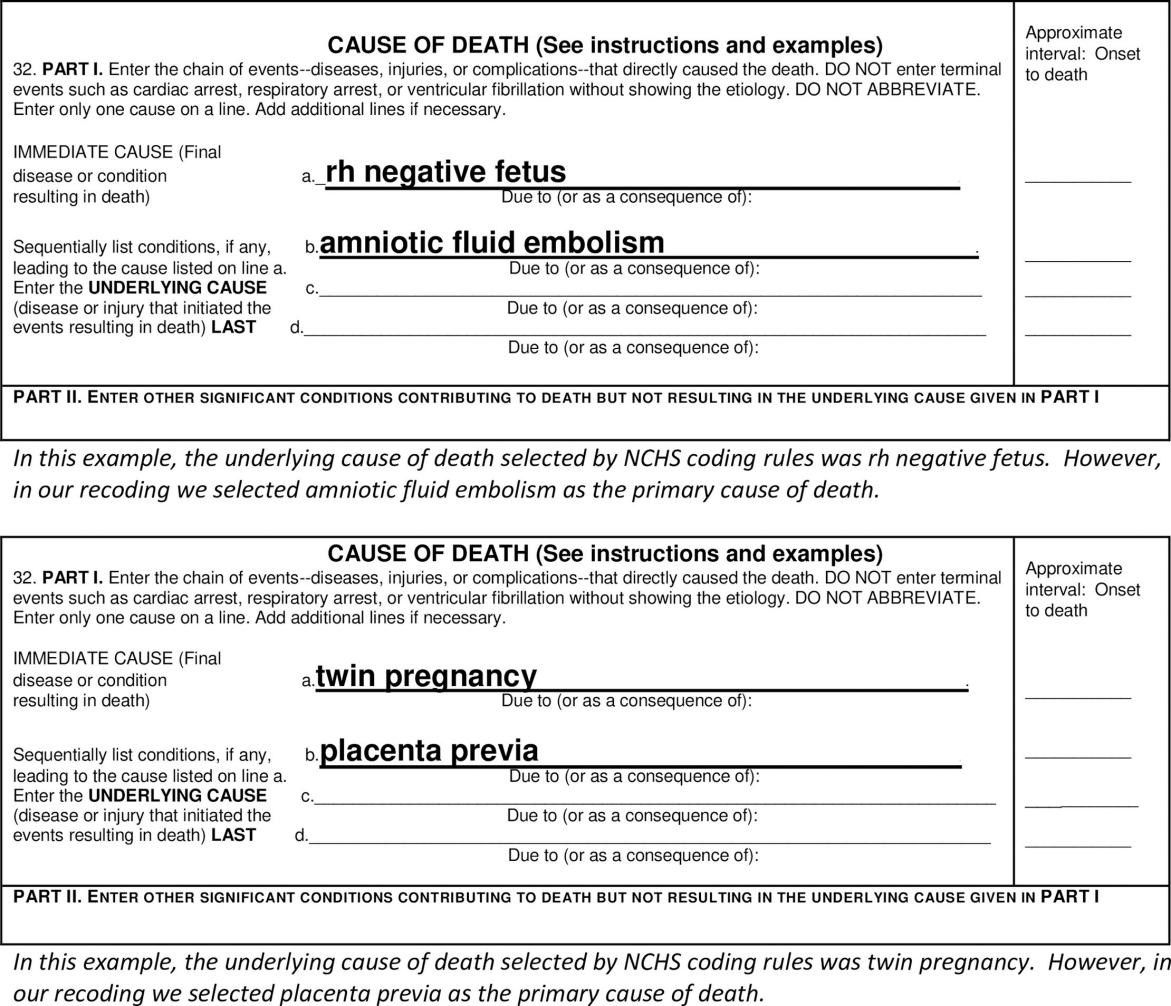
Examples of improper cause-of-death sequencing.

Standard NCHS underlying cause-of-death coding practices rely heavily on the *order* in which the several disease conditions on the death certificate are listed. They also rely heavily on the *sequence* of conditions–for example, whether one disease could have given rise to another disease (for example, cardiomyopathy leading to heart failure) or whether the diseases listed are unrelated (for example, diabetes and ectopic pregnancy) [17, 18]. These coding practices work well when death certificates have been properly completed, with correct sequencing and listing the underlying cause last. However, our initial review of the cause-of-death literals found that this was not the case for many maternal death certificates. When death certificates have not been properly completed, an application of the standard cause-of-death coding rules often does not result in the most informative cause of death being selected as the underlying cause (Fig 1).

We developed an alternative coding strategy to identify the *primary* cause of death directly from the literals. We defined the “primary cause of death” as the cause of death that was the most likely, or primary, cause that led to the decedent’s death, regardless of order listed on the death certificate [19]. In selecting the primary cause of death, we followed the underlying cause coding rules when applicable. However, we found cases where the cause written on the lowest used line (or occasionally in another location in the cause-of-death section) was clearly the most important and medically antecedent cause of death, but the sequence of causes was not medically plausible. In this case, we coded the most informative and medically antecedent cause as the primary cause of death. While assigning primary cause of death involves human judgment, we minimized bias by having all records jointly coded by two Ph.D. epidemiologists trained at NCHS (MM and MT). Any discrepancies between the coders were resolved via individual case review and discussion, reference to medical and coding texts [17, 18, 20–22], and consultation with medical and coding experts.

US coding practices specify that if the pregnancy checkbox indicates the death occurred during or within 1 year of pregnancy, and the death is due to natural causes (i.e. excluding accidents, homicide and suicide) then the cause of death is automatically coded as a maternal or late maternal death, regardless of whether the condition was related to or exacerbated by the pregnancy [18]. However, because of major problems with the pregnancy checkbox data [4, 8] we chose to examine each case independent of the checkbox. Thus, we recoded records with a pregnancy/postpartum mention in the cause-of-death literals as maternal deaths, and records with no such mention to non-maternal causes. This does not mean that the latter deaths were non-maternal, but merely indicates that we were unable to confirm the pregnancy status from the cause-of-death literals. The recoding was done to increase the specificity of conditions coded, as well as to provide an alternative code for cases where it was unclear whether or not they were maternal deaths.

The few codes available for classifying late maternal deaths (O96-O97) do not provide any information about the actual cause of death. Thus, late maternal deaths with pregnancy mention in the cause-of-death literals were coded to more specific maternal causes, while records with no such mention were coded to non-maternal causes.

### Analysis

We tabulated the recoded primary cause of death and compared it to the original NCHS reported underlying cause of death, hereafter referred to as original cause of death. In addition, we examined the primary cause of death specifically for the 43.5% of maternal death records originally coded to ill-defined causes (O26.8, O95, O99.8). For records without a pregnancy mention in the cause-of-death literals, unknown causes were recoded to R99 (cause unknown).

From a detailed examination of the literals, we developed a list of terms indicating pregnant/postpartum status (S1 Table) and created a variable to identify the presence or absence of this terminology. We compared pregnancy mentions in the literals by maternal age, maternal race and ethnicity, whether the person who certified the death was a physician, a medical examiner or coroner, and the timing of death within the pregnant/postpartum period. We also created a variable to identify concordance or discordance between the original and primary causes of death and the reasons for discordance. The study was ruled as exempt from IRB review by the University of Maryland IRB, because the study was based on death certificates and there were no living human subjects.

## Results

**Fig 2:**
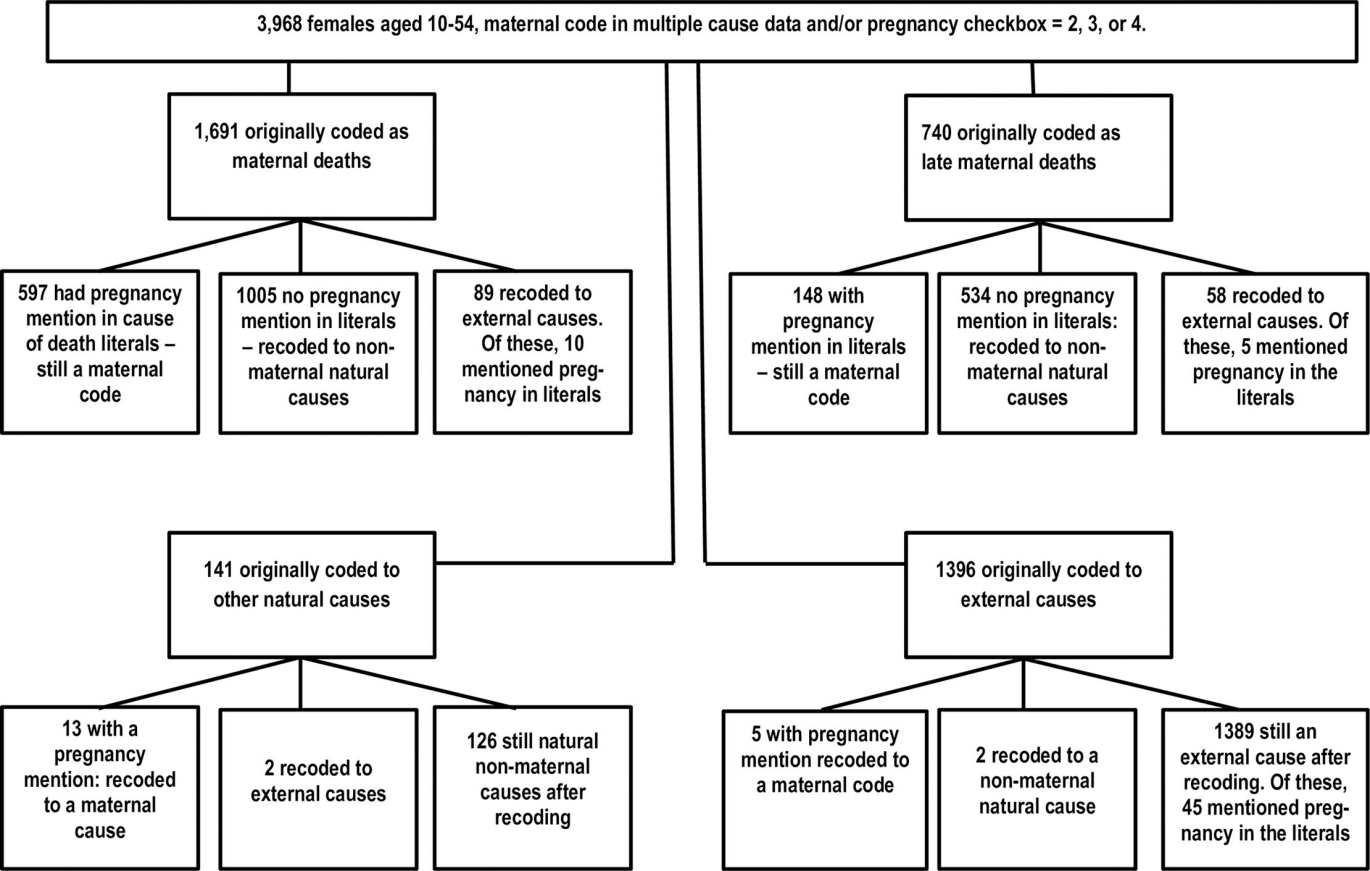
Flow chart for maternal mortality study, United States, 2016–2017.

Among the 3968 records selected for more detailed analysis, the original cause of death was as follows: 1691 maternal deaths (ICD-10 codes A34, O00-O95, O98-O99), 740 late maternal deaths (O96-O97), 141 other deaths coded to natural (non-maternal) causes (A00-N99, Q00-R99), and 1396 coded to external causes of injury (i.e accidents, homicide or suicide) (V01-Y98) (Fig 2).

Of the records originally coded as maternal deaths, 597 had a pregnancy mention in the cause-of-death literals and were confirmed as maternal deaths. 1005 had no pregnancy mention in the cause-of-death literals and were recoded to non-maternal natural causes because we were unable to verify their pregnant/postpartum status from the cause-of-death literals. An additional 89 records were recoded to external causes of death as the primary cause of death; of these, 10 mentioned pregnancy in the literals (Fig 2 –top left panel). Patterns were similar for late maternal deaths (Fig 2 –top right panel).

For 96.0% of records originally coded to non-maternal causes, the pregnancy checkbox provided the only evidence of pregnant/postpartum status (Fig 2 –bottom two panels). However, there were 18 records originally coded to non-maternal causes that we recoded to maternal causes due to a pregnancy mention in the literals, including 4 to postpartum cardiomyopathy, and 3 each to eclampsia/preeclampsia, complications of obstetric surgery and procedures, and obstetric embolism (which includes amniotic fluid embolism, pulmonary embolism, and any other type of embolism occurring during the pregnant/postpartum period). These records provide evidence of underreporting of maternal deaths due to coding issues. We selected the 1691 records originally coded as maternal deaths, and the 740 records originally coded as late maternal deaths for more detailed analysis.

### Primary cause of death: Records originally coded as maternal deaths

Of the 1691 records originally coded as maternal deaths, 597 (35.3%) had a pregnant/postpartum mention in the cause-of-death literals and were retained as maternal deaths (Table 1, columns 2 and 3). Among these, 418 (70.0%) retained the same code as the original cause-of-death assigned and 179 (30.0%) were reassigned to a more specific code. Among the 597 recoded maternal deaths, 544 (91.1%) were direct obstetric deaths. The most common causes in this group were hypertensive disorders, eclampsia and preeclampsia, obstetric hemorrhage, and obstetric embolism. Also within this group were 37 records (6.2%) coded to other complications of obstetric surgery and procedures (O75.4)–many of these involving complications during cesarean section. An additional 50 records (8.4%) were from indirect obstetric causes, 39 of which were coded to diseases of the circulatory system affecting pregnancy/postpartum.

**Table 1:** Records originally coded as maternal deaths and their recodes, United States, 2016–2017.

Cause of Death Categories (ICD-10)	Maternal deaths			
	Original codes, n = 1691	Recodes, n = 1691	Recode of records originally coded to ill-defined maternal causes (O26.8, O95, O98), n = 735	Late maternal deaths, n = 740
**Non-maternal causes**				
**Total coded to non-maternal causes**	**-**	**1094**	**622**	592
**Infections (A00-B99)**	**-**	**68**	**36**	**37**
Sepsis (A41.9)	**-**	48	30	21
**Cancer (C00-C97)**	**-**	**208**	**181**	**152**
Lung Cancer (C34.9)	**-**	25	22	9
Breast Cancer (C50.9)	**-**	45	42	36
Ovarian Cancer (C56)	**-**	18	17	4
**Endocrine, Nutritional, and Metabolic Diseases (E00-E90)**	**-**	**59**	**15**	**21**
Diabetes (E10-14)	**-**	46	6	13
**Diseases of the nervous system (G00-G99)**	**-**	**58**	**36**	**33**
Epilepsy/Seizures (G40)	**-**	31	18	18
**Diseases of the circulatory system (I00-I99)**	**-**	**340**	147	171
Hypertensive diseases (I10-I15)	**-**	27	2	17
Pulmonary embolism (I26.9)	**-**	51	20	34
Cardiomyopathy (I42)	**-**	26	15	16
Cardiac arrhythmias (I49)	**-**	17	4	17
Cardiovascular disease, unspecified (I516)	**-**	19	9	9
Intracerebral hemorrhage (I61)	**-**	25	8	7
Intracranial hemorrhage (I62.9)	**-**	21	15	4
**Diseases of the respiratory system (J00-J99)**	**-**	**67**	**43**	**44**
Pneumonia (J12-18)	**-**	34	23	23
**Diseases of the digestive system (K00-K93)**	**-**	**83**	**18**	**26**
Alcoholic cirrhosis of liver (K70.3)	**-**	19	1	2
**Diseases of the genitourinary system (N00-N99)**	**-**	**34**	**28**	**10**
Renal failure (N17-N19)	**-**	26	23	6
**Symptoms, signs and abnormal clinical and laboratory findings, not elsewhere classified (R00-R99)**	**-**	**36**	**31**	**16**
Other ill-defined and unspecified causes of mortality (R99)	**-**	27	26	14
**External causes of death (V01-Y98)**	**-**	**89**	**48**	**58**
Accidental poisoning, overdose (X40-X49)	**-**	66	35	43
**Maternal causes**				
**Total coded to maternal causes** (A34,O00-O95,O98-O99)	**1691**	**597**	**113**	**148**
**Obstetric death of unspecified cause (O95)**	**18**	**3**	**3**	**0**
**Total direct obstetric causes** (A34, O00-O92)	**1189**	**544**	**83**	**122**
Pregnancy with abortive outcome (O00-O07)	41	44	4	4
Ectopic pregnancy (O00)	27	32	3	1
Hypertensive disorders (O10-O16)	160	107	11	16
Pre-existing hypertension (O10)	53	12	1	2
Eclampsia and pre-eclampsia (O11,O13-O16)	105	95	10	14
Venous thrombosis in pregnancy (O22.0, O22.2,O22.3, O22.5,O22.9)	24	11	0	1
Obstetric hemorrhage (O20,O43.2,O44-O46,O67,O71.0, O71.1, O71.3,O71.4,O71.7,O72)	83	81	16	7
Pregnancy-related infection O23, O41.1, O75.3, O85, O86, O91)	39	35	3	3
Puerperal sepsis (O85)	17	13	0	3
Diabetes mellitus in pregnancy (O24)	48	8	0	1
Liver disorders in pregnancy (O26.6)	73	2	0	0
Other specified pregnancy-related conditions (O26.8)	476	11	11	0
Other complications of obstetric surgery and procedures (O75.4)	23	37	1	3
Obstetric embolism (O88)	92	95	23	15
Cardiomyopathy in the puerperium (O90.3)	63	82	11	68
**Total indirect causes** (O98-O99)	**484**	**50**	**27**	**26**
Mental disorders and diseases of the nervous system (O99.3)	37	2	1	0
Diseases of the circulatory system (O99.4)	148	39	18	14
Diseases of the respiratory system (O99.5)	20	4	4	1
Other specified diseases and conditions (O99.8)	241	1	1	6
**Total non-specific causes (O26.8, O95, O99.8, R99)**	735	42	41	20

Categories shown for causes of death with at least 1% of deaths in columns 2 or 3. ICD chapter titles only shown for chapters with specific categories shown under them. Residual categories are not shown to save space and promote clarity of presentation.

- category not applicable.

An additional 1094 records (64.7%) were recoded to non-maternal causes as their primary cause of death because we were unable to verify the woman’s pregnant/postpartum status from the cause-of-death literals. The exception to this was 10 records which had a pregnancy mention but were recoded to external causes of injury, which are excluded by the maternal mortality definition. Among the 1,094 records recoded to non-maternal causes, 31.1% (340) were recoded to diseases of the circulatory system, the most common of which were pulmonary embolism, intracerebral or intracranial hemorrhage, hypertensive diseases and cardiomyopathy. An additional 20.2% were recoded to cancers, the most common of which were breast, lung, and ovarian cancer. Other common causes of death for this group were sepsis, diabetes, pneumonia, and seizure disorders. Notably, 89 (8.1%) of these records were recoded to external causes of death; the majority of which (66) were due to accidental poisoning/overdose. Three cases were recoded to unknown cause (O95) which is neither a direct nor an indirect obstetric cause of death.

### Primary cause of death: Records originally coded to ill-defined maternal causes

Among the 1691 records originally coded as maternal deaths, 735 (43.5%) were originally coded to ill-defined or non-specific causes (O26.8, O95, O99.8) (Table 1, column 4). We were able to recode 694 (94.4%) of these cases to more specific causes of death as more specific information was available from the cause-of-death literals. Thus, only 41 records (5.6%) retained a non-specific cause code (O26.8, O95, O99.8, or R99) in our recoding. Among the 735 records originally coded to ill-defined causes, only 113 (15.4%) mentioned pregnant/postpartum status in the cause-of-death literals.

### Primary cause of death: Late maternal deaths

Among the 740 records originally coded as late maternal deaths (O96-O97), 148 (20.0%) had a postpartum mention in the cause-of-death literals (Table 1 column 5). Of these, 68 were recoded to postpartum cardiomyopathy, 16 to hypertensive disorders, 15 to obstetric embolism, and the remainder to other causes within the pregnancy chapter. For the remaining 592 (80.0%) deaths, we were unable to verify postpartum status from the cause-of-death literals, and these were recoded to non-maternal causes. The exception to this was 5 records which had a postpartum mention, but were recoded to external causes of injury which are excluded from the maternal mortality definition. Among the 592 deaths recoded to non-maternal causes, 171 (28.9%) were due to diseases of the circulatory system, the most common of which were pulmonary embolism, hypertensive disorders, cardiac arrhythmia and cardiomyopathy. Cancer accounted for an additional 152 deaths (25.7%). Also of note were 58 (9.8%) deaths recoded to external causes of injury, of which the majority (43) were due to accidental poisoning/overdose.

### Concordance and discordance between original and primary causes of death

Among the 1691 records originally coded as maternal deaths, 418 retained the same cause of death after recoding, and 1084 were recoded to non-maternal causes as the primary cause of death because we could not confirm pregnant/postpartum status from the cause-of-death literals (Table 2). The other discordant records were attributed to possible sequencing errors in how the death certificate was completed (n = 61), or other coding differences including records that were originally coded to non-specific causes that could be recoded to more specific causes based on information from the literals (n = 128). For the 740 records originally coded as late maternal deaths, 148 were recoded to more specific maternal causes, 586 were recoded to non-maternal causes, and 6 had other coding differences.

**Table 2:** Concordance and discordance between records originally coded as maternal and late maternal deaths and their recodes, and reasons for discordance, United States, 2016–2017.

Reason	Originally coded as maternal death, n = 1691	Originally coded as late maternal death, n = 740
**Code stayed the same**	418	0
**Checkbox only-no mention of pregnancy in literals: recoded to non-maternal causes**	1084	586
**Recoded due to problem with cause-of-death sequence**	61	0
**Other coding differences**	128	6
**Pregnancy mention in literals—recoded to more specific maternal causes**	0	148

### Differences by characteristics

The percentage of records with a pregnancy mention in the literals was much lower for women aged 45–54 (4.9%), when compared to women aged 10–39 (35–40%) (Table 3). Pregnancy mentions were more common among non-Hispanic black (36.7%) and Hispanic women (35.7%), than for non-Hispanic white women (25.1%). Records completed by physicians had a substantially lower percentage of cases with a pregnancy mention (23.4%), compared to records completed by a medical examiner or coroner (39.9%). There were also substantial differences by the timing of death within the pregnant/postpartum period. For women who were pregnant at the time of death, 22.7% had a pregnancy mention in the cause-of-death literals, compared to 40.8% for women who were < = 42 days postpartum, and 18.7% who were 43 days-1 year postpartum.

**Table 3:** Number and percentage of maternal and late maternal deaths with pregnancy mention in cause-of-death literals by selected variables, United States, 2016–2017.

	Pregnancy mentioned in cause-of-death literals, n (%)
**Total**	**760 (31.3)**
**Maternal age**	
<20	24(42.9)
20–24	110 (36.7)
25–29	180 (37.3)
30–34	179 (35.7)
35–39	178 (39.6)
40–44	69 (30.0)
45–54	20 (4.9)
**Race/ethnicity**	
Non-Hispanic white	285 (25.1)
Non-Hispanic black	284 (36.7)
Hispanic	141 (35.7)
Certifier*	
Physician	214 (23.4)
Medical examiner or coroner	217 (39.9)
Pregnancy checkbox**	
Pregnant at time of death	196 (22.7)
<42 days postpartum	288 (40.9)
43 days-1 year postpartum	135 (18.7)

*Certifier was not reported for 38.3% of records, and these cases were dropped before percentages were computed.

**Pregnancy checkbox values of 1 (not pregnant within past year), 8 (not on certificate) and 9 (unknown if pregnant within past year) were not analyzed separately since, by definition, records with those values were only included in the subset if pregnancy was mentioned in the cause-of-death literals.

## Discussion

This paper addressed three key challenges in the recording of maternal deaths in the U.S.: (1) the influence of the pregnancy checkbox in coding maternal deaths; (2) the large proportion of deaths coded to ill-defined causes; and (3) the lack of a specific cause of death for late maternal deaths. Among the 1691 maternal deaths reported in vital statistics data, just over 1/3 (35.9%) mentioned pregnancy or postpartum status in the cause-of-death literals. This indicates that a substantial majority (64.7%) were originally coded as maternal deaths solely due a checkbox entry. Among these records, the most common recoded causes of death were diseases of the circulatory system and cancer. This distribution by cause of death was similar to all-cause mortality for women of reproductive age, once external causes of death (which by definition are excluded from maternal deaths) are omitted [23]. For late maternal deaths, we found that 80% were coded as maternal solely based on the pregnancy checkbox.

The failure to provide an indication of pregnancy in the cause-of-death literals does not necessarily mean the death was non-maternal, but rather indicates that we could not verify the woman’s pregnancy status from the literals. Still, the large number of such cases together with the similarity of causes of death with those of reproductive age women suggests that some of these cases may not be maternal deaths at all, but were classified as such due to errors in the pregnancy checkbox. Conversely, the indication through the pregnancy checkbox combined with verification in the literals, should provide considerable confidence that these are maternal deaths.

Fully 43.5% of the originally coded maternal deaths were coded to ill-defined or non-specific causes. More specific cause-of-death information was almost universally available from literal text for these cases, and we were able to recode 94.4% to more specific causes. We recommend that NCHS reevaluate their coding of non-specific causes to reduce the number of maternal deaths coded to these uninformative causes.

Consistent with other studies, we found a lower proportion of death certificates that mentioned pregnancy for women over 45 [9, 24], and for death certificates completed by physicians compared to medical examiners/coroners [8]. Findings by timing of death and race/ethnicity are relatively recent [24, 25], and need confirmation in future studies.

We found evidence of underreporting of maternal deaths in the 18 deaths which were originally coded to non-maternal causes, but which mentioned pregnancy in the literals and were recoded to maternal causes in our study. Additional quality control measures to examine pregnancy terminology within the literal text could decrease underreporting of maternal deaths.

Validation studies to confirm a woman’s pregnant/postpartum status can also benefit from examining the cause-of-death literals. For a substantial proportion of women, examination of the cause-of-death literals is a relatively cost-effective way to confirm a woman’s pregnancy or postpartum status with a high degree of certainty. This could substantially reduce the number of cases where recontacting the certifier is needed to confirm the decedent’s pregnancy status, thus decreasing the workload for state vital statistics offices and maternal mortality review committees in confirming maternal deaths.

### Strengths and limitations

The strengths of this study include the use of cause-of-death literals to systematically investigate all possible maternal deaths identifiable from death certificates in the United States in 2016–17. Using literal text, we identified more specific and detailed information on the circumstances surrounding death, which was often lost in cause-of-death coding. Our purpose was to analyze a cohort of *confirmed* maternal deaths; still we recognize that our methods may underestimate the number of maternal deaths if the woman’s pregnancy or postpartum status was not mentioned in the cause-of-death literals. It is also possible that the authors made errors in coding the primary cause of death. We minimized this possibility through careful review and discussion of each identified case. When the determination of a case was unclear, we consulted with coding and medical experts, and additional reference materials such as WHO and NCHS ICD-10 coding manuals [17, 18], medical textbooks and medical dictionaries [20–22] to confirm diagnoses. Finally, our inclusion criteria could still result in misascertainment of maternal deaths if the physician, medical examiner, or coroner who filled out the death certificate did not accurately report the cause of death.

## Conclusions

Beginning with 2018 data, NCHS has once again begun publishing official maternal mortality statistics for the United States, after an 11-year hiatus [6]. In doing this, they have made two changes to how they identify maternal deaths, including restricting application of the pregnancy checkbox to decedents aged 10–44 years (due to decreased checkbox accuracy for women 45–54), and restricting assignment of maternal codes to the underlying cause alone (not multiple causes of death) when the checkbox is the only indication of pregnancy [6]. It is a tremendous step forward for the United States to again be publishing official maternal mortality statistics, and these changes have the potential to improve reporting of maternal deaths to a certain extent. However, our study has shown that a majority of vital statistics maternal deaths are identified by the pregnancy checkbox alone, which has been proven to be unreliable in multiple studies. Thus, applying an after-the-fact algorithm or fix is no substitute for doing the quality control and training up front to improve the accuracy of the pregnancy checkbox information. National, state and local systems involved in death certification need to better train physicians, medical examiners and coroners on the importance of the pregnancy checkbox and completing the cause of death section. Establishment of internal consistency checks in real time and changes to cause-of-maternal-death coding procedures could significantly improve reporting. The recent interest of policymakers [26] and the media [27] in maternal mortality, the US’s poor international ranking [28, 29], and the many preventable maternal deaths in the US each year [30], lend urgency to the development of more accurate and detailed maternal mortality data.

## Supporting information

S1 Table Pregnancy-related terms reported on death certificates, United States, 2016–2017. (DOCX)
