# Supplementary material for: Improving US maternal mortality reporting by analyzing literal text on death certificates, United States, 2016–2017
Source: PLoS One. 2020 Oct 28;15(10):e0240701. doi: 10.1371/journal.pone.0240701 (PMC7592741; doi:10.1371/journal.pone.0240701)
Supplement: S1 Table — (DOCX) [file pone.0240701.s001.docx]

| **S1 Table. Pregnancy-related terms reported on death certificates, United States, 2016-2017** |
| --- |
| Eclampsia (Echlampsia) |
| Preeclampsia (Pre-eclampsia, Pre-clampsia, Pre-eclampsion, pre eclampsa) |
| Gestation (Gestational) |
| Pregnancy (Pregnant) |
| Postpartum (Post-partum, Post-partal, Post-entram, Post partum, Post par-tum, Post parturition) |
| HELLP |
| Cesarean (C-section, Csection, C section, Caesarean, Emergency section) |
| Ectopic |
| Abortion (Induced termination) |
| Peripartum (Para-partum, Peri-partum) |
| Trimester |
| Gravidarum |
| Gravid |
| Labor |
| Delivery* |
| Chorioamnionitis (Chorio) |
| Birth* |
| Placenta (Placental) |
| Amniotic |
| Uterine rupture* (Rupture of uterus, Ruptured uterus) |
| Uterine atony* |
| Childbirth* |
| Intrauterine* |
| Umbilical |
| Accreta (Percreta, Increta) |
| Epidural* |
| Maternal* |
| Fetal* (Fetus) |
| Trophoblast (Trophoblastic) |
| Stillbirth |
| Miscarriage |
| Puerperal (Purpura, Puerpera, Puerperium) |
| Parentheses indicate iterations of similar terminology found on the death certificate, including misspellings. |
| * Indicates terminology that requires additional review to confirm pregnancy-related status. |
